# Donor Evaluation Tool: A New Technology Improves Donor Enrolment on ICU

**DOI:** 10.3389/ti.2024.12227

**Published:** 2024-07-26

**Authors:** Chiara Imbimbo, Marcus Nauwerk, Tizian Cammarota, Franziska Beyeler, Nathalie Krügel, Andreas Elmer, Thomas F. Mueller, Franz Immer

**Affiliations:** Swisstransplant, National Foundation for Organ Donation and Transplantation, Bern, Switzerland

**Keywords:** digital, ICU, organ assessment, tool, organ evaluation

## Abstract

Uncertainties on the intensive care unit (ICU) regarding the eligibility of a patient to be a potential deceased organ donor may prevent their referral and enrolment in the pathway for organ donation. Healthcare staff may exclude potential donors for medical reasons, which are no longer applicable. Hence, Swisstransplant implemented a digital donor evaluation tool (DET) in 2021, which allows the local hospital’s organ donation coordinator to send a direct request to medical advisors (MA) of the organ procurement organization before excluding potential donors. All 156 requests entered in 2022 were analyzed. 117 patients (75.0%) were primarily accepted by the MA as potential donors. Of those 60 patients (51.3%) became actual organ donors. Main reasons for using the DET were questions regarding malignancies (n = 33, 21.2%), infectious diseases (n = 35, 22.4%) and age/co-morbidities (n = 34, 21.8%). The average age of the actual “DET donor” compared to the regularly enrolled, actual “Non-DET donor” was 65.3 ± 15.8 vs. 56.8 ± 17.5 years, respectively (*p* = 0.008). On average 1.9 ± 1.1 organs compared to 3.2 ± 1.3 organs were retrieved from DET vs. Non-DET donors. In summary, this new digital donor evaluation tool supports reporting and facilitates eligibility decisions in uncertain, complex donor cases, potentially increasing the number of organ donations.

## Graphical Abstract

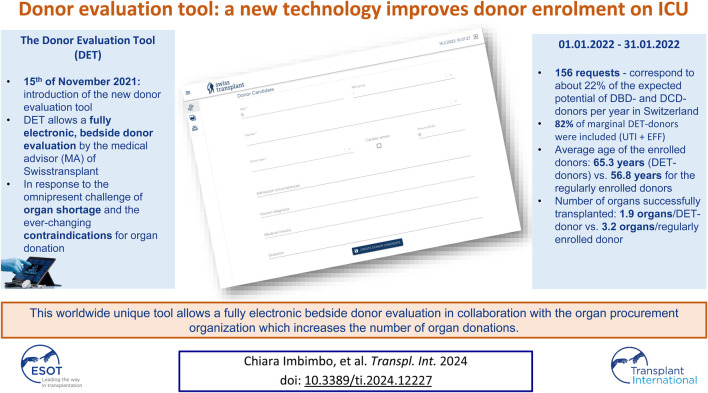

## Introduction

Organ transplantation from deceased donors is a well-established medical treatment and very often the only curative therapeutical option in advanced organ failure. Organ shortage represents an omnipresent challenge in transplantation medicine worldwide. Switzerland is no exception with a post-mortem organ donation rate of 18.3 donors per million people (pmp) in 2019 [1]. In comparison, other European countries such as Spain or France register considerably higher numbers with post-mortem donor rates of 49.6 and 29.4 pmp, respectively [1]. Facing this issue Switzerland has implemented different approaches in the past. As in various other countries, donations after circulatory death (DCD) have been introduced and there has been an increased use of expanded-criteria donors over time [2–4].

In addition, national programs have been launched aiming to raise public awareness and improve structures, resources and processes at the hospital level [5]. Despite the great efforts undertaken, the demand for donor organs continues to exceed the current supply by far resulting in extensive waiting periods and higher death rates on the waiting list [6]. These global realities mandate that novel approaches are urgently needed.

Over the years, the exchange between hospitals and Swisstransplant, the National Foundation for Organ Donation and Transplantation, has become more and more intensive, especially recently due to the COVID-19 pandemic. During this challenging time Switzerland followed a gradual shutdown-approach with the aim to prevent the transplantation activity from collapsing. A centralized evaluation of all potential organ donors was implemented and performed by medical advisors (MA) within Swisstransplant [7] with a special focus on factors such as availability of resources, organ quality and urgency status of the recipients on the National Waiting List. As a result, the number of transplantations performed remained almost unaffected despite the comparably high Covid-case load at that time [8].

This positive experience formed the basis for developing a digital platform to facilitate hospitals directly contacting Swisstransplant’s MAs, in case of uncertainty regarding the suitability of a patient for organ donation and the further procedure. Eligibility criteria for organ donation are constantly updated and modified by new knowledge, so their application is not always easy to the multi-factorial cases of complex donors. Hence, guidelines for organ donation are regularly reviewed and adjusted. Most of the ICU staff is not involved in these discussions and potential donors may be excluded for reasons, which are no longer applicable. Moreover, enrollment of marginal donors may also depend on the medical urgency and profile of actual recipients on the National Waiting List. In critical urgent patients transplant centers are willing to take a higher risk in accepting marginal organs.

On the 15th of November 2021 Swisstransplant implemented a digital donor evaluation tool (DET), which allows the hospital’s organ donation coordinator, informed by the ICU staff, to fill out a donor evaluation form and send a request to the medical advisors (MA) from Swisstransplant in any case of uncertainty regarding suitability for donation before excluding potential donors. Based on the medical condition of the potential donor and the situation on the National Waiting List the MA gives electronically written feedback to the requesting center.

The present article describes the effects of using the DET in the first year.

## Materials and Methods

This study analyzes all requests sent via the DET in 2022, the first calendar year after its introduction on 15th November 2021.

The provincial ethics committee (KEK) granted exemption for the underlying study (BASEC-no. Req-2024-00085). For this kind of retrospective study approval is not required according to the Swiss human research law (Humanforschungsgesetz, Art. 2, Abs. 1).

**FIGURE 1:**
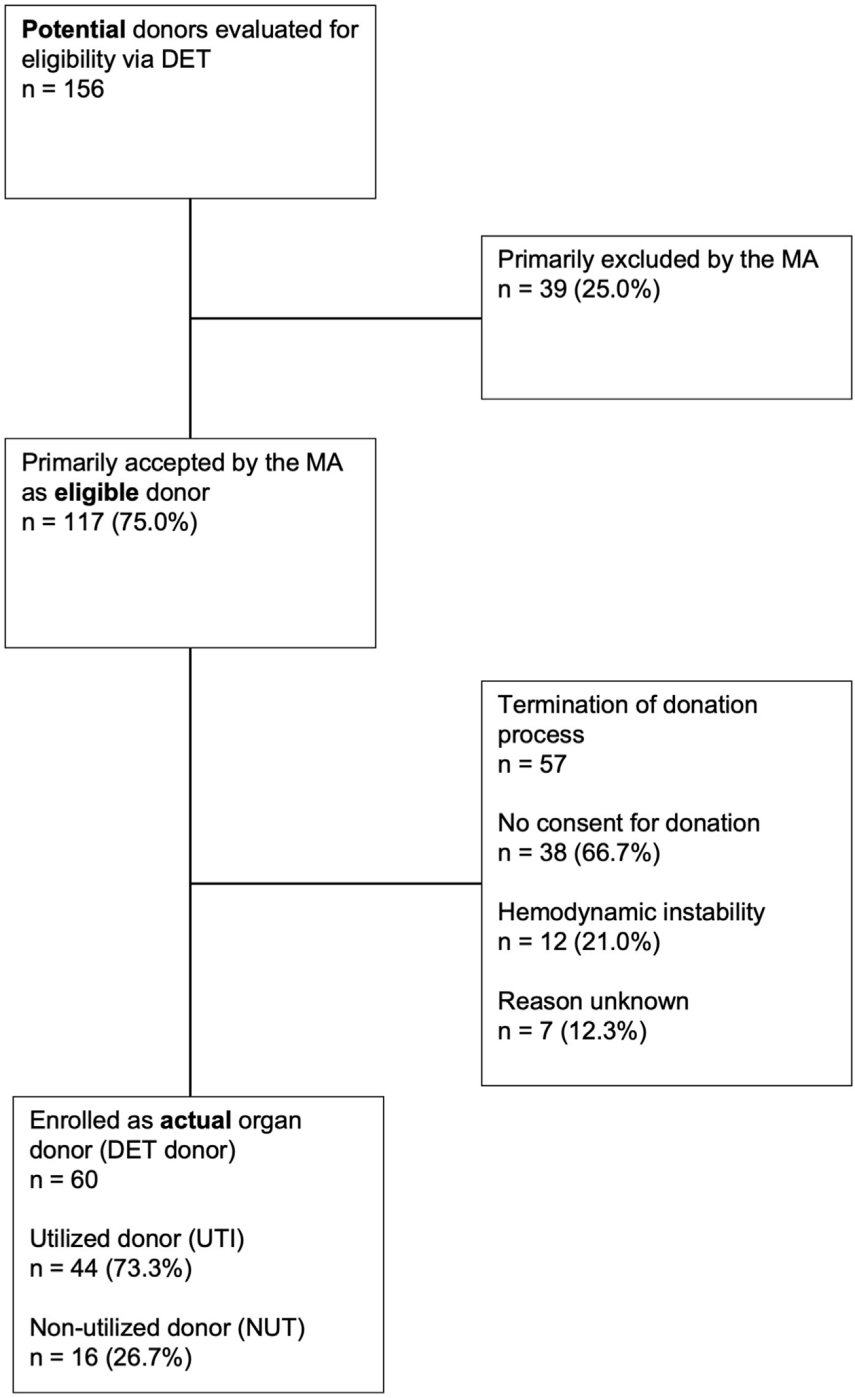
Flowchart of all requests digitally submitted to the medical advisors via the donor evaluation tool (DET) Overview of course and final outcome of all 156 requests submitted in 2022 to the medical advisors (MAs) at Swisstransplant via the donor evaluation tool (DET). UTI, utilized donor; NUT, non-utilized donor.

On behalf of Swisstransplant the company *isolutions AG, Berne, Switzerland*, programmed this application, which enables a fast and digital exchange with the hospitals. In the supplement a link and QR-code is provided showing a video of the practical application of the DET. The aim of this work was to analyze the outcome of all digitally entered requests for evaluation. The decision process is shown in Figure 1 and based on the nomenclature of the critical pathway for deceased donation [9, 10]. The assessment of the DET requests by the Swisstransplant MAs could lead to either direct exclusion or primary acceptance as medically suitable donor. The consecutive work-up could lead to either termination of the donation process or enrollment and registration as actual organ donor in the Swiss Organ Allocation System (SOAS), i.e., declared dead, eligible and consented for organ donation. These registered, actual donors were subcategorized into (a) “utilized donor” (UTI), i.e., at least one organ was transplanted and (b) “non-utilized donor” (NUT), i.e., no organ was recovered or transplanted.

The group of utilized donors enrolled through DET (DET donors) was then compared with the group of utilized donors enrolled through the standard, regular direct registration (Non-DET donors) in the Swiss Organ Allocation System (SOAS) during the same period of time. Donations of preceding years before installment of the DET system were not included due to annual variabilities in numbers, substantial effects of the Covid pandemic and changes in donor acceptance criteria over time.

The two groups of DET and Non-DET donors were compared with descriptive statistics using Fisher’s exact test for categorical values (cause of death groups), two sample t-test for continuous variables (age and body mass index), and Pearson’s Chi-squared test for categorical data (sex and donor categories).

## Results

### Number of Requests Using DET

A total of 156 requests of individual patient cases were entered in the DET in 2022 (see also Figure 1, flowchart). This corresponds to approximately 22% of the estimated potential of DBD (donation after brain death) and DCD (donation after cardiocirculatory death) donors on ICUs per year in Switzerland according to the database of SwissPOD (Swiss Monitoring of Potential Donors). In this database all deaths on ICUs in Switzerland are recorded by Swisstransplant as required by law.

117 patients (75.0% of the total 156 requests) were primarily accepted as eligible donors on the initial assessment by the MA. Out of these 117 eligible donors 60 (51.3%) were ultimately enrolled as actual organ donors. In the remaining 57 (48.7%) patients no consent for organ donation was the main cause (n = 38, 66.7%) for stopping the donation process. This resulted in a refusal rate of around 33% of eligible donors within the DET group and is thus notably lower than the Swiss average of approximately 55% [11]. In another 12 cases (21.0%) donation was not possible due to the further clinical course with hemodynamic instability of the patient or findings in additional investigations. In the remaining 7 cases (12.3%), the reason for not donating could not be determined retrospectively.

### Reason for Using DET

The analysis of the 156 enquiries identified three main concerns leading to the use of the digital evaluation tool: infectious diseases (n = 35, 22.4%), malignancies (n = 33, 21.2%) and old age and/or various co-morbidities (n = 34, 21.8%). Issues related to SARS-CoV-2 infections accounted for a large proportion of the infection-related queries. Out of the 35 infection-related requests 17 cases (49%) were related due to a diagnosis of SARS-CoV-2 infection.

### Comparison of Donors Enrolled With DET vs. Standard Enrollment (Non-DET)

**FIGURE 2:**
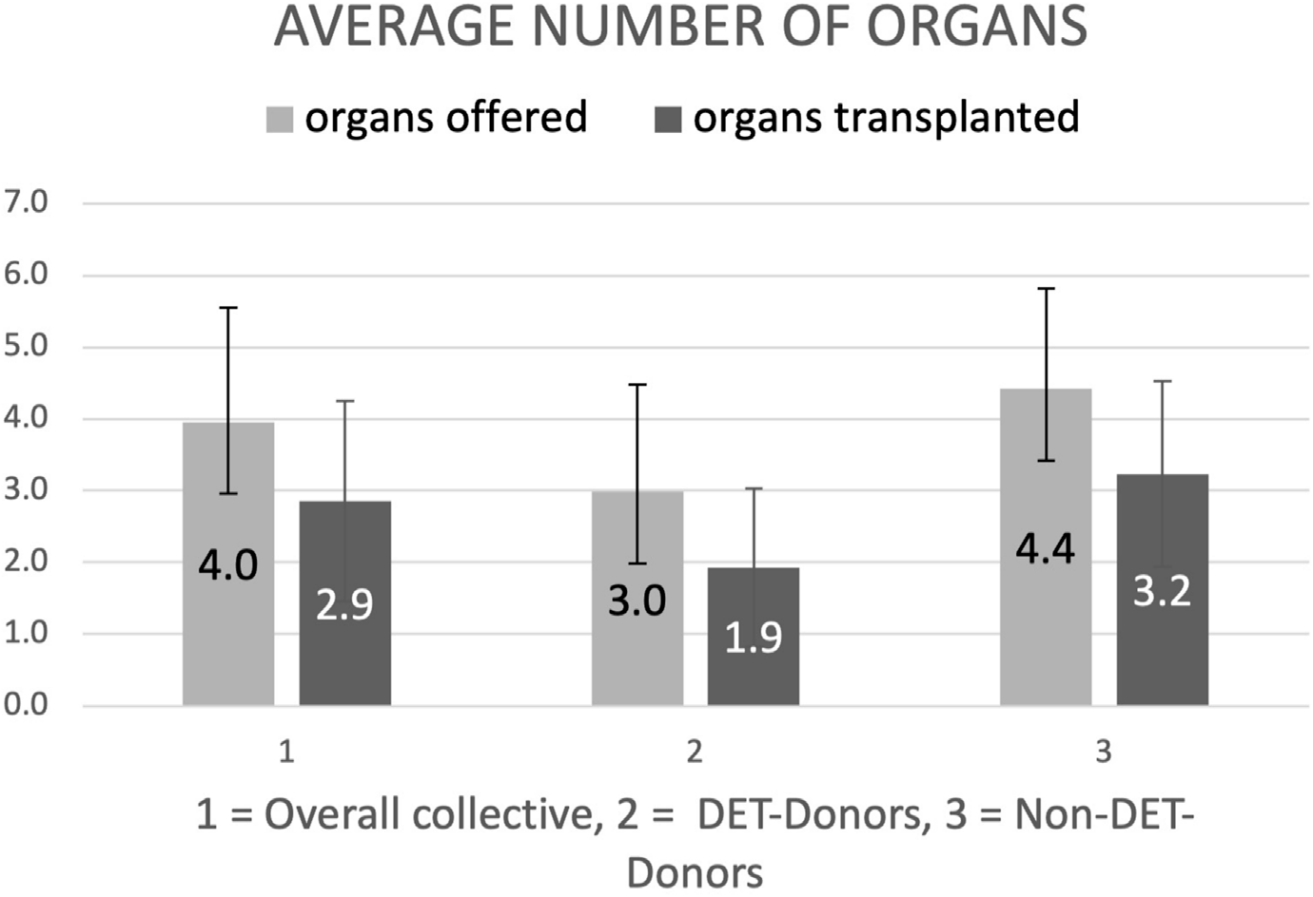
Average number of organs offered (light grey bars) and transplanted (dark grey bars) of donors enrolled with the donor evaluation tool (DET donors) and enrolled with the standard registration process (Non-DET donors). Results in mean ± standard deviation (SD) organs per donor.

As shown in Table 1 the evaluation of the patients’ characteristics showed a significantly higher age of the actual DET donors compared to the actual Non-DET donors (65.3 ± 15.8 vs. 56.8 ± 17.5 years, resp., *p* = 0.001). In total 73.23% of the DET donors became utilized (UTI) donors, compared to 89.7% of the Non-DET donors (Figure 2).

**TABLE 1:** Comparison between donors enrolled with the donor evaluation tool (DET donors) and with the standard registration process (Non-DET donors).

		DET donors		Non-DET donors		
		n	%	n	%	*p*-value
Donors		60	32.3	126	67.7	0.004^b^
	UTI	44	73.3	113	89.7	
	NUT	16	26.7	13	10.3	
Age^a^		65.3	15.8	56.8	17.5	0.001^c^
Sex						0.87^b^
	Male	36	60	74	58.7	
	Female	24	40	52	41.3	
Type						0.41^b^
	DBD	29	48.3	69	54.8	
	DCD	31	51.7	57	45.2	
BMI^a^		28.8	4.6	26.9	5.3	0.013^c^
COD						0.19^d^
	Anoxia	27	45	48	38.1	
	Cerebral hemorrhage	19	31.7	50	39.7	
	Cerebral trauma	6	10	17	13.5	
	Cerebral disease	2	3.3	8	6.3	
	Cerebral tumor	1	1.7	0	0	
	Other	4	6.7	2	1.6	

DET, digital evaluation tool; UTI, utilized donor; NUT, non-utilized donor; DBD, donation after brain death; DCD, donation after cardiocirculatory death; BMI, body mass index; COD, cause of death.

^a^ mean (SD).

^b^ Pearson’s Chi-squared test.

^c^ Two sample t-test.

^d^ Fisher’s exact test.

Regarding gender distribution, both groups showed a largely equal distribution with a male share of around 60%. The main causes of death for both cohorts were anoxic brain damage (DET donors: 45%, Non-DET donors: 38%) and cerebral hemorrhage (DET donors: 32%, Non-DET donors: 40%).

In total, 98 (52.7%) DBD and 88 (47.3%) DCD donations were reported in 2022. The latter cold all be subclassified as controlled Maastricht III donors. Within the DET group 48.3% (n = 29) were DBD and 51.7% (n = 31) DCD donations, compared to the Non-DET donors, in whom the ratio of DBD to DCD donations was higher with 54.8% (n = 69) to 45.2% (n = 57).

**FIGURE 3:**
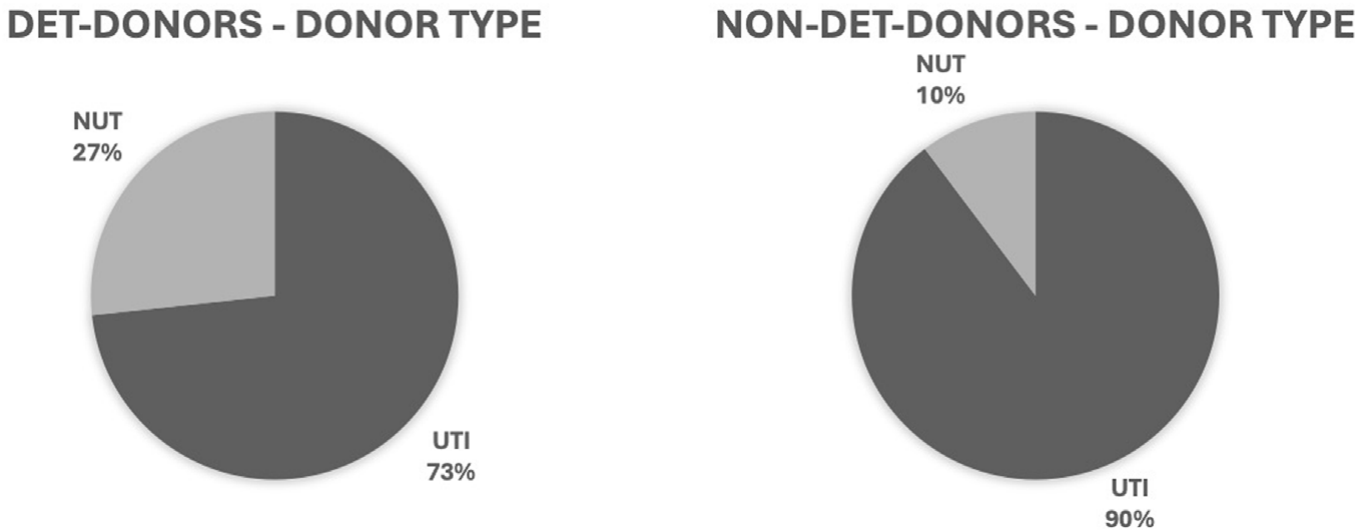
Distribution of donor types enrolled with the donor evaluation tool (DET donors) and with the standard registration process (Non-DET donors). Results in relative frequency (%). UTI, utilized donor; NUT, non-utilized donor.

On average, 1.9 ± 1.1 organs were transplanted per DET donor, compared to 3.2 ± 1.3 organs per Non-DET donor (Figure 3). Also the average number of offered organs were higher in the Non-DET compared to the DET donors (4.4 ± 1.4 vs. 3.0 ± 1.5, resp.).

## Discussion

### Key Factors of the Donor Evaluation Tool (DET)

For an organ to be successfully transplanted, a complex, time-consuming and labor-some process consisting of various steps must be completed beforehand. It starts with the identification of potential donors and the clarification of their suitability for donation. It includes a professional approach towards the relatives and specialized medical management to ensure the quality of the donation process. Each of these steps poses various challenges for the medical professionals involved and, if not handled properly, can lead to the loss of potential donors and ultimately of transplantable organs.

Based on the experience during the COVID-pandemic with a centralized evaluation, it became apparent that there is a potential to enroll more marginal donors for organ donation and also that knowledge of the current waiting list is of great importance in order to make decisions on donor eligibility.

The support of ICU staff in the assessment of medical suitability for donation is of increasing importance, in particular due to the progress of new findings justifying more liberal inclusion criteria for potential donors. Swisstransplant established the so-called “Donor Evaluation Tool” (DET) in November 2021 to offer hospitals a digital solution for quickly and directly contacting the specialist on duty in the event of uncertainty regarding the eligibility of a potential donor. Compared to the standard telephone contact, the tool offers the advantage to upload important documents such as laboratory values or radiology reports from the patient’s medical file in addition to the mandatory information such as age, gender, or suspected donation type (see also online video). This provides the MA directly with an information package facilitating the primary decision of principal eligibility of the patient as organ donor.

The analysis of the first year 2022 since the introduction of the DET suggests that direct accessibility of a specialist advisor, here the MA of Swisstransplant, and his or her consecutive expert evaluation especially regarding complex and marginal potential organ donors might be a resource to increase the number of organ donors to those regularly enrolled and registered.

The potential added value of the DET pathway is likely multi-factorial. The MA is an expert in the field of organ donation and transplantation who is constantly learning about the latest international developments and findings. Thus, the MA supports the ICU staff with the highly specialized expertise in the assessment of complex cases regarding organ donation. Another decisive factor is the knowledge regarding the situation on the waiting list, which is then also considered by the MA in the evaluation process. In critical urgent patients transplant centers take a higher risk in accepting marginal organs and hence the referral rate by ICU specialists of cases that otherwise might be lost might be higher.

### Main Concerns: Transmission of Malignancies and Infections

The key uncertainties on behalf of the ICU staff leading to the use of the DET were the potential risk that the donor might transmit an infectious or malignant disease.

Various studies have shown, that the risk of transmission of a malignant disease is always present, but overall classified as rather low. Studies from the United Kingdom (UK) indicated an overall risk of transmitting a malignant disease of about 0.05% [12], the risk of transmission by donors with a known history of malignancy of 1.1% [13]. A study in the US reached comparable results, 650 organs were transplanted from 257 deceased donors with a history of cancer. In the follow-up period of 45 months, none of the respective organ recipients developed a cancer of the original donor type [14].

Similar findings were published for the risk of transmission of infectious diseases. The “Ad Hoc Disease Transmission Advisory Committee (DTAC)” recorded 2,185 potential disease transmission events, of those only 15% (335 donors) were classified as proven/probable donor-derived diseases, including 244 transmitted infections and 70 malignant diseases. Despite overall rare, however, diseases transmitted by organ donation have a high morbidity and mortality and prevention strategies or approaches for early detection are necessary [15]. Overall, these studies indicate the need for specialist evaluation and reassurance of the treating physicians regarding infectious or malignant diseases in potential donors. They also explain why more than 40% of requests submitted via the DET to the specialist advisor were related to tumors and infections in potential donors.

Around 20% of requests were related towards age and/or comorbidities. This again indicates uncertainties of ICU staff regarding the eligibility of marginal donors, in particular as guidelines cannot provide clear-cut age-thresholds or disease exclusions. Here again the direct accessibility to an experienced MA overseeing the changing trends in donor/recipient criteria, special organ characteristics and waitlist demands is helpful [10].

### Comparable Approaches in Other Countries

Experiences with a centralized evaluation of organ donors have so far only been described in the literature from Israel and Italy. Cohen and Ashkenazi analyzed the number and type of enquiries received over a period of 10 years since the introduction of a centralized “medical advisory service” (MAS) in Israel in 2007. Hospitals can call a specialist at any given time to discuss questions regarding the organ donation process. Concerns regarding the safety of organs for transplantation, especially in case of malignant or infectious donor diseases, were the main reason for enquiries to the MAS. The authors concluded that such a model would be a valuable tool to increase the number of donor organs as well as safety, quality and standardization of the donation process [16].

In 2003, Italy established a similar system on a national level with a continuously available expert task force. However, enquiries are limited to an evaluation of potential donors with a possible risk of transmission of an infectious or malignant disease. Nevertheless, the application of uniform guidelines and the expert evaluation and risk assessment also achieved a higher number of organs for transplantation [17].

These results from Israel and Italy are in line with the experiences made in Switzerland with the DET.

### The Added Value of the DET Pathway to the Standard Process of Donor Enrolment

It is difficult to quantify the added effect of the newly implemented DET pathway in this retrospective study. In particular long-term data and comparable granularity of information for both pathways require prospective, future studies.

However, the requests put in the DET system indicate that the ICU staff needs help in the evaluation of marginal donors, i.e., complex patients with comorbidities and advanced age. In these cases, the central decision by a MA to proceed or not with the donation process is not only medically reassuring but likely also helpful for the staff to proceed with the laborious and emotionally demanding process of donor work-up, including family involvement, additional diagnostic tests, and extended stay on the ICU. In this context, it is very reassuring that out of the primarily accepted eligible donors 50% became actual donors. In addition, the positive impact of the DET process is underlined by the roughly 20% lower consent refusal rate compared to the Swiss national average rate. In addition, the digital submission of key data together with the question facilitates decision making for the MA.

As expected for a marginal donor population the DET patients were nearly 10 years older than those enrolled via the standard registration. However, despite the lower donor utilization rate of 74% vs. 90% and less organs transplanted of 1.9 vs. 3.2 for the DET vs. Non-DET donors, respectively, these numbers indicate the added value of the tool and justify the continuation of this service to the ICU staff. Future studies have to analyze the outcome data for DET vs. Non-DET donors and whether the total numbers of organs transplanted has significantly increased due to the DET pathway.

### Limitations of the Study

Some limitations need to be mentioned. Robust numbers regarding a definite increase of donors due to DET cannot be given. The annual variability of donation rates due to effects such as initiation of DCD donation or COVID pandemics would require a longer period of observation. For example, in 2022 a substantial number of questions were related to COVID infections (11%). These inquiries might drop in the future. In addition, it is unclear whether all donors submitted and finally utilized via DET would ultimately have been missed without the digital tool. It is possible that the ICU-physician together with the responsible coordinator would have alternatively used the standard way of reporting for these potential donors or sought advice by other means and experts in the field.

Nevertheless, according to the current figures, the frequency of requests shows an increasing trend. This likely reflects the broad acceptance of the DET in the hospitals across the country.

Another limitation is the variability of information content given in the individual enquiries. For a comprehensive evaluation in regard to organ donation and for a better prospective analysis of the additional impact of the DET pathway, a further standardization of the mandatory electronic data input and a learning curve on both sides, ICU hospitals and organ procurement organization, will improve the performance of tool and its efficiency. In addition, another goal is to improve also information granularity and standardization in the Non-DET pathway which will allow then also a better comparison of both processes, in particular an in-depth analysis of phenotypes of DET vs. Non-DET donors. Addressing these early limitations will likely turn into additional strengths of the future applications of DET.

Overall, future prospective analyses will be necessary to further evaluate the impact of this new tool on donation rates as well as long-term postoperative transplant outcomes.

## Conclusion

This retrospective study analyzes the first full calendar year (2022) of using a unique donor evaluation tool (DET). This electronic device allows direct and easy access for the ICU staff in case of uncertainty regarding eligibility of a potential donor. In addition the fully electronic donor evaluation facilitates also the decision making by the medical expert. This facilitated enquiry and evaluation process might increase deceased donor numbers but more long-term data and prospective studies are needed.

## Data Availability

The raw data supporting the conclusions of this article will be made available by the authors, without undue reservation.
