# Supplementary figures and images for: Donor Evaluation Tool: A New Technology Improves Donor Enrolment on ICU
Source: Transpl Int. 2024 Jul 26;37:12227. doi: 10.3389/ti.2024.12227 (PMC11310011; doi:10.3389/ti.2024.12227)

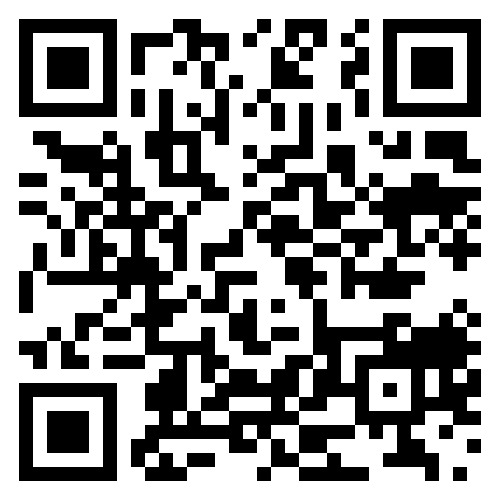

Supplement: Supplementary file 1 [file Image1.JPEG]
